# Phenotyping data coupled with RNA sequencing of apple genotypes exhibiting contrasted quantitative trait loci architecture for apple scab (*Venturia inaequalis*) resistance

**DOI:** 10.1016/j.dib.2024.110778

**Published:** 2024-07-31

**Authors:** Juliette Bénéjam, Julie Ferreira de Carvalho, Elisa Ravon, Christelle Heintz, Matthieu Gaucher, Charles-Eric Durel, Marie-Noëlle Brisset, Laure Perchepied

**Affiliations:** University of Angers, Institut Agro, INRAE, IRHS, SFR QUASAV, F-49000 Angers, France

**Keywords:** *Malus domestica*, QTL, Transcriptomic, Genetic resistance mechanisms, Biotic interactions

## Abstract

Previous studies have highlighted the role of three quantitative trait loci (QTL, i.e. ‘qT1’, ‘qF11’ and ‘qF17’) in partial resistance to apple scab. Underlying molecular mechanisms of these loci are yet unknown. Exploring differential gene expression between apple genotypes carrying contrasting combinations of these QTLs could depict original candidate genes and pathways implicated. We therefore carried out RNA sequencing just before and five days after inoculation of the pathogenic fungi *Venturia inaequalis*, in sixteen genotypes from a pseudo-F1 progeny segregating for resistant or susceptible alleles of the three QTLs. The current dataset includes i) transcriptomic profile description, ii) analysis of differentially expressed genes related to none or combined QTLs, infected or not with *Venturia inaequalis* and iii) disease phenotyping of the same genetic materials. The raw data files have been deposited in the Gene Expression Omnibus (GEO) repository with the accession number GSE250309. These outputs represent the first step towards elucidating the genetic basis of quantitative apple scab resistance. In the long term, this data set will improve apple breeding strategies on how to combine qualitative (used so far) and quantitative resistances to apple scab, with the aim of diversifying selective pressures on the pathogen.

Specifications Table

**Table:** 

Subject	Plant Science: Plant Microbe Interaction
Specific subject area	Transcriptomics and genetic resistance to biotic stress
Type of data	Tables and figures Raw phenotypic data (.csv), filtered raw reads (fastq), analysed RNA-seq data files (raw_counts.sf), table (xls) and figures (pptx)
Data collection	Data were obtained from young potted apple trees composed of scions carrying four contrasted QTL architecture for apple scab resistance, grafted on the rootstock MM106 and artificially inoculated with *Venturia inaequalis*. Phenotypic data were obtained by scoring the disease progression 14-, 21- and 28-days post inoculation (dpi). Total RNA was extracted from leaves collected at 0 and 5 dpi. RNA sequencing was performed by Genome Quebec (Canada, https://genomequebec.com/), using the Illumina NovaSeq 6000 S4 PE100 technology to generate 100 bp pair-end sequencing. The Area Under the Disease-Progress Curve or AUDPC was calculated over the three scoring dates. Processing of RNA-seq data included (1) raw reads filtering, (2) paired-reads mapping onto the *Malus domestica* GDDH13 reference transcriptome [1], and (3) bioinformatic analysis for differential gene expression.
Data source location	Institution: Research Institute for Horticulture and Seeds (IRHS), University of Angers, Institut Agro, INRAE City/Town/Region: 49071 Beaucouzé Country: France Latitude and longitude for collected samples/data: Not available
Data accessibility	*The raw and processed RNA-seq data were deposited at the Gene Expression Omnibus (GEO) database with the accession number GSE250309. Phenotypic data are included in this article.* *Repository name: Gene Expression Omnibus (GEO) database* *Data identification number: Accession number GSE250309* *Direct URL to data:*https://www.ncbi.nlm.nih.gov/geo/query/acc.cgi?acc=GSE250309
Related research article	[2] *J. Bénéjam, E. Ravon, M. Gaucher, M.-N. Brisset, C.-E. Durel,* L. *Perchepied, Acibenzolar-S-Methyl and resistance Quantitative Trait Loci complement each other to control apple scab and fire blight, Plant Dis. 105 (2021) 1702–1710.*https://doi.org/10.1094/PDIS-07-20-1439-RE.

## 1 Value of the Data

• These data contribute to the understanding of the molecular basis of quantitative apple scab resistances.
• Knowing the molecular mechanisms underlying QTLs can assist further research in the field of breeding by pyramiding sources of resistance that act differently, in order to improve resistance sustainability.
• Our transcriptomic dataset can be used for identifying novel genes and pathways involved in apple response to *Venturia inaequalis.*

## 2 Background

The three QTLs for resistance against apple scab were previously identified in different studies ([[3], [4], [5], [6], [7]]). Located on LG (Linkage Group) 1 of the apple hybrid TN10-8 (QTL ‘qT1’) and on LG11 and LG17 of the apple cultivar Fiesta (QTLs ‘qF11’ and ‘qF17’, respectively), their genetic positions were recently refined [2] thanks to a F1 progeny of a cross between TN10-8 and Fiesta (‘TxF progeny’) described in [8]. ‘qT1’ co-localized with the *Rvi6* (*Vf*) gene at position 43.03 cM of the genetic map corresponding to the CH-Vf1 simple sequence repeat marker, tightly associated to that R-gene [9]. ‘qT1’ is thus a potential allele or paralog of *Rvi6* with quantitative resistance effect while ‘qF11’ and ‘qF17’ could instead be involved in a signaling or downstream defense pathway ([2,7]). To further understand the molecular mechanisms underlying these QTLs, we designed an experiment to generate phenotypic and RNA-seq data from four classes of genotypes of the ‘TxF progeny’ carrying either: 1) no resistance QTL alleles among ‘qT1’, ‘qF11’ and ‘qF17’ (referred after as ‘NoScabQTL’), 2) a resistance allele at ‘qT1’ only (‘qT1’), 3) resistance alleles at both ‘qF11’ and ‘qF17’ (‘qF11qF17’) and 4) resistance alleles at the three QTLs (‘qT1qF11qF17’).

## 3 Data Description

**Fig. 1:**
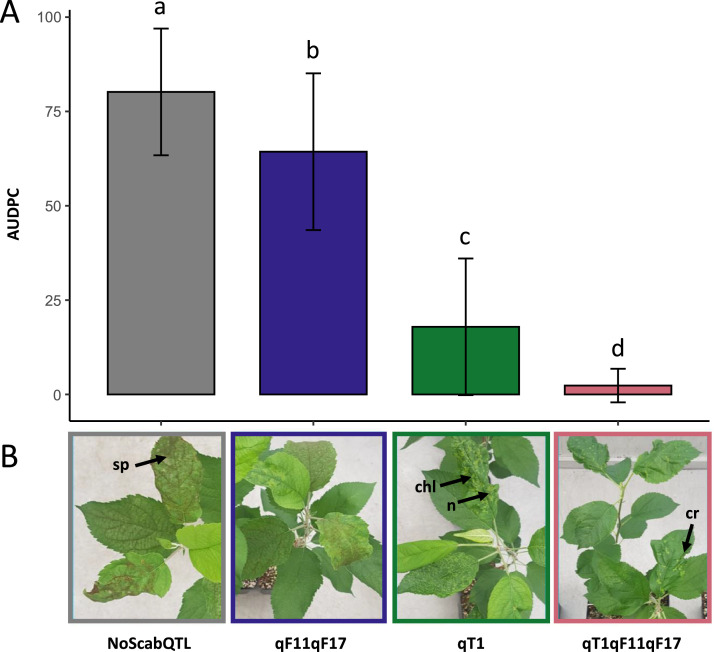
Comparison of symptoms between classes of QTL after *V. inaequalis* inoculation. A) AUDPC of leaf scab severity (% sporulating lesions). Letters indicate groups showing significant differences according to HSD test. B) Representative disease (sporulating lesions (sp)) or resistance (crispation of leaf (cr), chlorosis (chl) and necrosis (n)) symptoms for each class of genotypes, 28 days after inoculation.

The comparison of symptom development between the four classes of QTL after artificial inoculation by *V. inaequalis* can be visualized in Fig. 1, with the Area Under the Disease-Progress Curve (AUDPC) calculated over the three scoring dates (Fig. 1A) and an illustration of the disease and resistance symptoms recorded (Fig. 1B). The Supplemental Table 1 contains the AUDPC values for each genotype representing the four classes of QTL.

Transcriptomic data for each class of QTL just before (0 dpi) and 5 days (5 dpi) after inoculation were obtained by RNA sequencing using an Illumina NovaSeq 6000 S4 PE100 platform. Output reads ranged from 29.3 to 41 million bases per sample and mapping rates from 85.7 % to 87.2 % (Table 1). The original sequencing datasets have been deposited in the Gene Expression Omnibus (GEO) with the accession number GSE250309.

**Table 1:** Results of sequencing and mapping.

RNA sample	Class of QTL	Sampling day (dpi)	Replicate	Total bases (Mb)	Mapping rate (%)
1	NoScabQTL	0	1	41.03	86.04
2	NoScabQTL	0	2	33.88	85.89
3	qT1	0	1	38.10	85.80
4	qT1	0	2	40.37	85.94
5	qF11qF17	0	1	39.29	87.12
6	qF11qF17	0	2	38.79	86.29
7	qT1qF11qF17	0	1	31.50	86.34
8	qT1qF11qF17	0	2	36.93	87.17
9	NoScabQTL	5	1	38.68	86.96
10	NoScabQTL	5	2	29.70	86.22
11	qT1	5	1	36.55	85.74
12	qT1	5	2	33.68	86.31
13	qF11qF17	5	1	36.56	86.52
14	qF11qF17	5	2	29.33	86.72
15	qT1qF11qF17	5	1	35.60	86.29
16	qT1qF11qF17	5	2	38.10	85.91

**Fig. 2:**
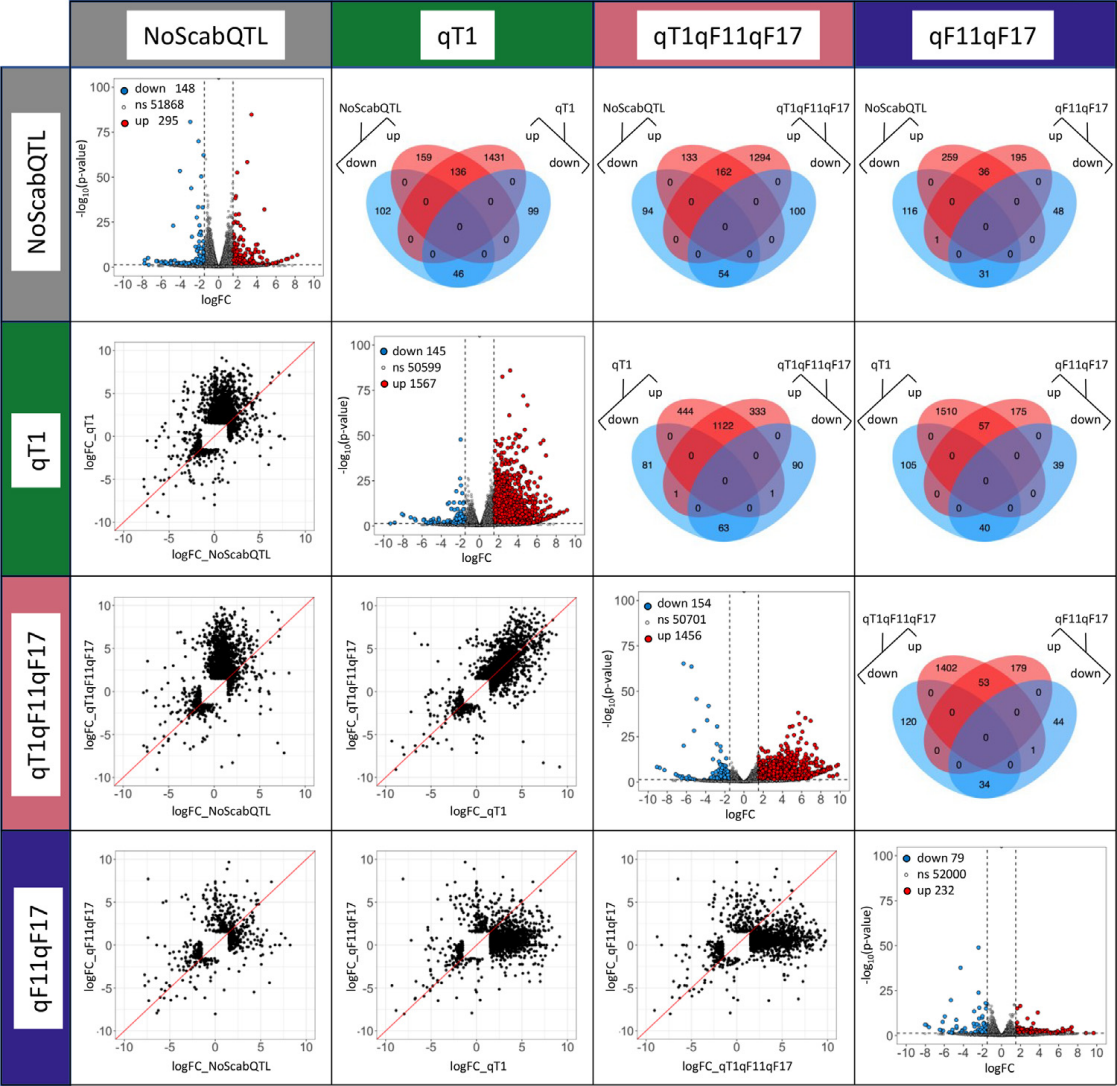
DEGs related to infection by *V. inaequalis* in each class of QTL. On the diagonal: volcano plots (5 dpi vs. 0), on the left of the diagonal: scatterplots of each class of QTL plotted against each other, on the right of the diagonal: Venn diagrams of specific and common DEGs between classes of QTL.

Differentially expressed genes (DEGs) related to infection (5 versus 0 dpi) in each class of QTL are illustrated in Fig. 2. Volcano plots display the logFC (log2 fold change) against the −log_10_ P value for each class of QTL. Scatterplots and Venn diagrams compare the DEGs between classes of genotypes two by two.

**Fig. 3:**
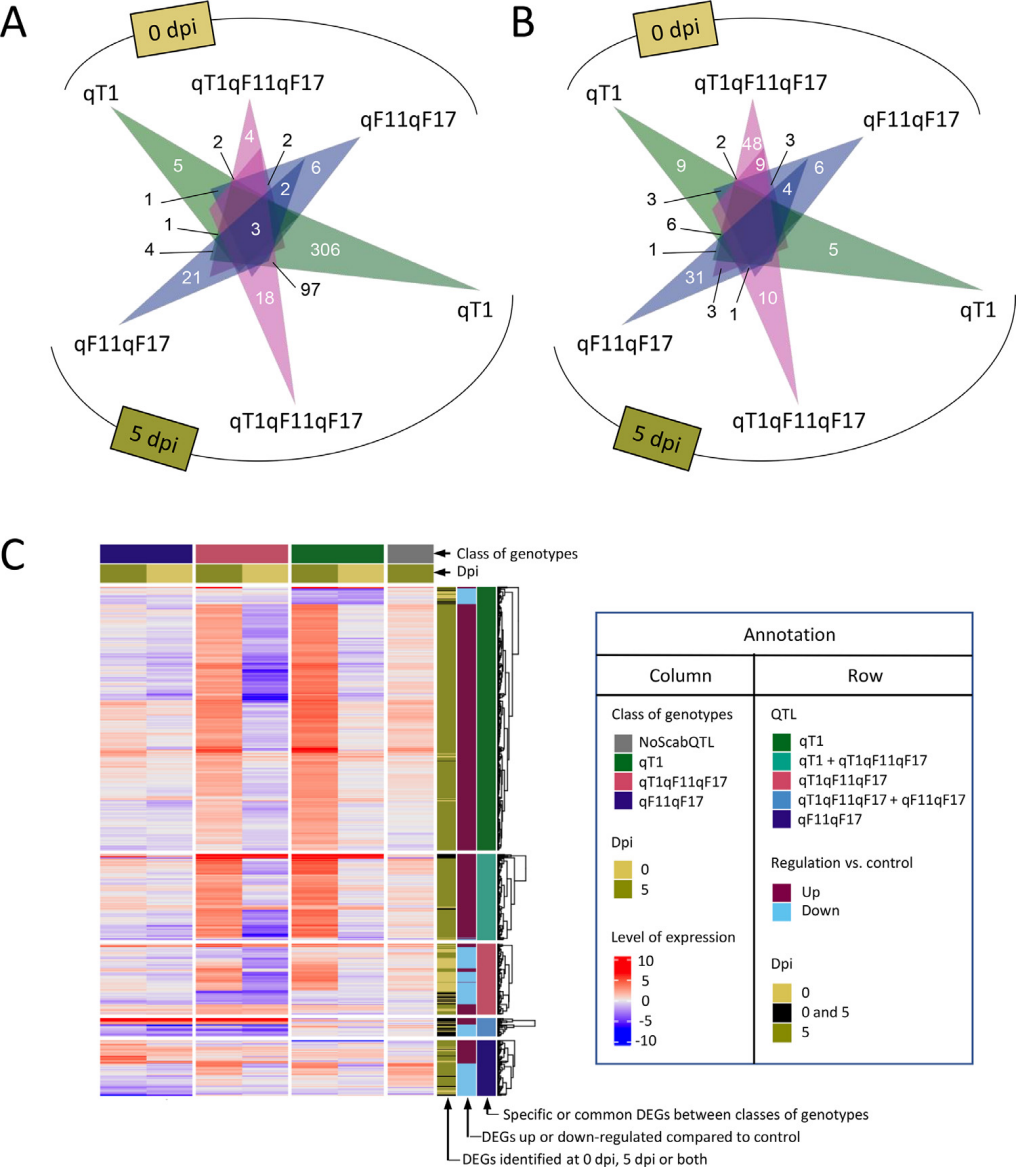
DEGs related to the presence of QTLs. A and B) Venn diagrams illustrating the overlap among differentially up- (A) or down-regulated (B) genes due to the presence of QTLs, when comparing each sampling time-point to ‘NoScabQTL’. C) Heatmap generated with the logFC values in each class of QTL at each sampling time-point vs. ‘NoScabQTL’ at 0 dpi and split by columns according to the presence of QTL and by rows according to the Venn results.

DEGs related to the presence of QTLs (‘qX’ versus ‘NoScabQTL’) at each sampling timepoint are illustrated in Fig. 3. Venn diagrams are used to draw common and specific up- or down-regulated DEGs in the three classes of genotypes carrying one QTL at least (Fig.3A and B). A heatmap was generated based on logFC values of these DEGs (except those common between ‘qT1’ and ‘qF11F17’) between each class of genotypes at 0 or 5 dpi and ‘NoScabQTL’ at 0 dpi (Fig. 3C).

## 4 Experimental Design, Materials and Methods

### 4.1 Plant material, growth conditions and inoculation

Each of the four contrasting classes of QTL was composed of four genotypes: F025, F138, F193 and F233 for the control class; F064, F117, F207 and F244 for the ‘qT1’ class; F135, F139, F171 and F336 for the ‘qF11qF17’ class; F072, F073, F081 and F259 for the ‘qT1qF11qF17’ class (adapted from [8]). Scions were collected from a conservatory orchard located at the Institut National de Recherche pour l'Agriculture, l'Alimentation et l'Environnement (INRAE, Angers, France) and grafted on MM106 rootstock (up to ten replicates per genotype). Young trees were grown in randomized blocks in greenhouse at INRAE Angers (France) under semi-controlled growing conditions (23 °C day/20 °C night, humidity 40 to 80 %, and artificial light to complement natural light). The inoculation was performed on actively growing shoots using the reference isolate ‘EU-B04’ of *V. inaequalis* (Origin: Belgium, host: Golden Delicious) previously described in [11] and [12]. Monoconidial suspension was prepared from diseased dry leaves at a concentration of 2.5 × 10^5^ conidia.ml^−1^ and sprayed on the young trees, transiently incubated 2 days at 17 °C under a plastic sheet to maintain a high humidity according to the conditions described by Caffier et al. [10].

### 4.2 Phenotypic assessment and data analysis

The percentage of leaf surface exhibiting sporulating lesions was scored at 14-, 21- and 28-days post-inoculation using the ordinal scale (0 to 7 from [13]) described in [3]. After filtering raw file for mistakes and missing data, the AUDPC over the three scoring dates was calculated. An ANOVA was applied using the package ‘aov’ of R software [14] to evaluate the effect of the classes of QTL and to compare the AUDPC variation between each class. The Tukey post-hoc test was performed using the package ‘HSD.test’. A boxplot was produced using the packages ‘ggpubr’ and ‘rstatix’ to summarise the results.

### 4.3 Leaf sampling RNA extraction/sequencing and transcriptomic analysis

Half of the two youngest leaves per plant were collected from two replicates of each genotype at each sampling time point, pooled, directly frozen in liquid nitrogen and stored at −80 °C. The same sampling procedure was performed on two other replicates of each genotype at each sampling time point in order to get a second biological replicate per genotype and sampling time point. Leaf samples were ground in liquid nitrogen and total RNA extractions were performed using the kit Nucleospin® RNA Plant (Macherey-Nagel GmbH & Co., Düren, Germany). For each biological replicate and sampling time point, same amounts of RNA from the four genotypes per class of QTL were pooled in order to get a total of 16 RNA samples. RNA quality and concentration were checked using a Bioanalyzer (Agilent Technologies, Santa Clara, CA, USA). Libraries were generated using the Illumina mRNA Stranded protocol and sequenced with the Illumina NovaSeq 6000 S4 PE100 reads technology (Génome Québec, Canada). The sequenced reads were mapped on the reference transcriptional units from GDDH13 v1.1 using Salmon software [15]. Transcript levels were calculated in CPM and DEGs were defined as exhibiting at least 1.5-fold change in transcript abundance and statistical levels of *p* < 0.05 relative to a control, using DESeq2, including a Benjamini–Hochberg procedure to control the false discovery rate (FDR) [16]. Venn diagrams were drawn according to Bardou et al. (2014) [17] and volcano-, scatter- and heatmap-plots generated using ggplot2 and ComplexHeatmap R package (https://cran.r-project.org/package=ggrepel, [18,19]).

## Limitations

None.

## Ethics Statement

The authors have read and followed the ethical requirements for publication in Data in Brief and confirm that the current work does not involve human subjects, animal experiments, or any data collected from social media platforms.

## CRediT Author Statement

**Bénéjam Juliette:** Data curation, Roles/Writing - original draft. **Ferreira de Carvalho Julie:** Writing - original draft/review/editing. **Ravon Elisa:** Data curation. **Heintz Christelle:** Data curation. **Gaucher Matthieu:** Methodology. **Durel Charles-Eric:** Conceptualization (phenotypic data), Funding acquisition, Writing - review & editing. **Brisset Marie-Noëlle:** Conceptualization, Formal analysis, Funding acquisition, Writing - original draft/review/editing. **Perchepied Laure:** Conceptualization, Data curation, Formal analysis, Writing - original draft/review/editing.

## Data Availability

Modulation of apple leaf transcriptome by QTLs and infection by Venturia inaequalis (Original data) (Gene Expression Omnibus).

## Appendix Supplementary materials

Image, application 1
