# Supplementary material for: Phenotyping data coupled with RNA sequencing of apple genotypes exhibiting contrasted quantitative trait loci architecture for apple scab (Venturia inaequalis) resistance
Source: Data Brief. 2024 Jul 31;56:110778. doi: 10.1016/j.dib.2024.110778 (PMC11374964; doi:10.1016/j.dib.2024.110778)
Supplement: Supplementary file 1 [file mmc1.docx]

**Appendix. Supplementary materials**

**Supplemental Table 1**.  AUDPC values and classification of the genotypes according to the different classes.

| Class of QTL | Genotype ID | Replicate | | AUDPC |
| --- | --- | --- | --- | --- |
| NoScabQTL | F025 | rep1 | 91.5 | |
| NoScabQTL | F025 | rep2 | 102 | |
| NoScabQTL | F025 | rep3 | 102 | |
| NoScabQTL | F025 | rep4 | 91.5 | |
| NoScabQTL | F025 | rep5 | 65 | |
| NoScabQTL | F025 | rep6 | 68 | |
| NoScabQTL | F025 | rep7 | 74.5 | |
| NoScabQTL | F025 | rep8 | 58.5 | |
| NoScabQTL | F025 | rep9 | 81 | |
| NoScabQTL | F025 | rep10 | 98 | |
| NoScabQTL | F025 | rep11 | 81 | |
| NoScabQTL | F025 | rep12 | 98 | |
| NoScabQTL | F025 | rep13 | 82 | |
| NoScabQTL | F025 | rep14 | 78 | |
| NoScabQTL | F193 | rep1 | 68 | |
| NoScabQTL | F193 | rep2 | 85 | |
| NoScabQTL | F193 | rep3 | 91.5 | |
| NoScabQTL | F193 | rep4 | 65 | |
| NoScabQTL | F193 | rep5 | 15.5 | |
| NoScabQTL | F193 | rep6 | 58.5 | |
| NoScabQTL | F193 | rep7 | 87.5 | |
| NoScabQTL | F193 | rep8 | 71.5 | |
| NoScabQTL | F193 | rep9 | 102 | |
| NoScabQTL | F193 | rep10 | 81 | |
| NoScabQTL | F193 | rep11 | 81 | |
| NoScabQTL | F193 | rep12 | 62 | |
| NoScabQTL | F193 | rep13 | 91.5 | |
| NoScabQTL | F193 | rep14 | 88.5 | |
| NoScabQTL | F233 | rep1 | 87.5 | |
| NoScabQTL | F233 | rep2 | 91.5 | |
| NoScabQTL | F233 | rep3 | 81 | |
| NoScabQTL | F233 | rep4 | 78 | |
| NoScabQTL | F233 | rep5 | 81 | |
| NoScabQTL | F233 | rep6 | 87.5 | |
| NoScabQTL | F138 | *NA* | *NA* | |
| qF11qF17 | F135 | rep1 | 55.5 | |
| qF11qF17 | F135 | rep2 | 71.5 | |
| qF11qF17 | F135 | rep3 | 37.5 | |
| qF11qF17 | F135 | rep4 | 65 | |
| qF11qF17 | F135 | rep5 | 71.5 | |
| qF11qF17 | F135 | rep6 | 51.5 | |
| qF11qF17 | F135 | rep7 | 91.5 | |
| qF11qF17 | F135 | rep8 | 6 | |
| qF11qF17 | F135 | rep9 | 51.5 | |
| qF11qF17 | F135 | rep10 | 71.5 | |
| qF11qF17 | F139 | rep1 | 48.5 | |
| qF11qF17 | F139 | rep2 | 62 | |
| qF11qF17 | F139 | rep3 | 22 | |
| qF11qF17 | F139 | rep4 | 62 | |
| qF11qF17 | F139 | rep5 | 68.5 | |
| qF11qF17 | F171 | rep1 | 74.5 | |
| qF11qF17 | F171 | rep2 | 58.5 | |
| qF11qF17 | F171 | rep3 | 31.5 | |
| qF11qF17 | F171 | rep4 | 85 | |
| qF11qF17 | F171 | rep5 | 68 | |
| qF11qF17 | F171 | rep6 | 71.5 | |
| qF11qF17 | F171 | rep7 | 71.5 | |
| qF11qF17 | F171 | rep8 | 75.5 | |
| qF11qF17 | F171 | rep9 | 88.5 | |
| qF11qF17 | F171 | rep10 | 71.5 | |
| qF11qF17 | F171 | rep11 | 88.5 | |
| qF11qF17 | F171 | rep12 | 62 | |
| qF11qF17 | F171 | rep13 | 91.5 | |
| qF11qF17 | F171 | rep14 | 91.5 | |
| qF11qF17 | F336 | *NA* | *NA* | |
| qT1 | F064 | rep1 | 15.5 | |
| qT1 | F064 | rep2 | 0 | |
| qT1 | F064 | rep3 | 55.5 | |
| qT1 | F064 | rep4 | 25 | |
| qT1 | F064 | rep5 | 35.5 | |
| qT1 | F064 | rep6 | 42 | |
| qT1 | F064 | rep7 | 0 | |
| qT1 | F064 | rep8 | 19 | |
| qT1 | F064 | rep9 | 40 | |
| qT1 | F064 | rep10 | 22 | |
| qT1 | F064 | rep11 | 22 | |
| qT1 | F064 | rep12 | 3 | |
| qT1 | F064 | rep13 | 6 | |
| qT1 | F064 | rep14 | 9.5 | |
| qT1 | F117 | rep1 | 6.5 | |
| qT1 | F117 | rep2 | 0 | |
| qT1 | F117 | rep3 | 0 | |
| qT1 | F117 | rep4 | 0 | |
| qT1 | F117 | rep5 | 6 | |
| qT1 | F117 | rep6 | 19 | |
| qT1 | F117 | rep7 | 15.5 | |
| qT1 | F117 | rep8 | 0 | |
| qT1 | F117 | rep9 | 0 | |
| qT1 | F117 | rep10 | 43 | |
| qT1 | F117 | rep11 | 6 | |
| qT1 | F117 | rep12 | 28.5 | |
| qT1 | F117 | rep13 | 3 | |
| qT1 | F117 | rep14 | 6 | |
| qT1 | F207 | rep1 | 59 | |
| qT1 | F207 | rep2 | 59 | |
| qT1 | F207 | rep3 | 22 | |
| qT1 | F207 | rep4 | 0 | |
| qT1 | F207 | rep5 | 22 | |
| qT1 | F207 | rep6 | 19 | |
| qT1 | F244 | *NA* | *NA* | |
| qT1qF11qF17 | F072 | rep1 | 0 | |
| qT1qF11qF17 | F072 | rep2 | 0 | |
| qT1qF11qF17 | F072 | rep3 | 0 | |
| qT1qF11qF17 | F072 | rep4 | 0 | |
| qT1qF11qF17 | F072 | rep5 | 0 | |
| qT1qF11qF17 | F072 | rep6 | 0 | |
| qT1qF11qF17 | F072 | rep7 | 0 | |
| qT1qF11qF17 | F072 | rep8 | 0 | |
| qT1qF11qF17 | F072 | rep9 | 3 | |
| qT1qF11qF17 | F072 | rep10 | 0 | |
| qT1qF11qF17 | F072 | rep11 | 0 | |
| qT1qF11qF17 | F072 | rep12 | 3 | |
| qT1qF11qF17 | F072 | rep13 | 0 | |
| qT1qF11qF17 | F081 | rep1 | 0 | |
| qT1qF11qF17 | F081 | rep2 | 6.5 | |
| qT1qF11qF17 | F081 | rep3 | 0 | |
| qT1qF11qF17 | F081 | rep4 | 0 | |
| qT1qF11qF17 | F081 | rep5 | 0 | |
| qT1qF11qF17 | F081 | rep6 | 0 | |
| qT1qF11qF17 | F081 | rep7 | 0 | |
| qT1qF11qF17 | F081 | rep8 | 0 | |
| qT1qF11qF17 | F081 | rep9 | 3 | |
| qT1qF11qF17 | F081 | rep10 | 0 | |
| qT1qF11qF17 | F081 | rep11 | 12.5 | |
| qT1qF11qF17 | F081 | rep12 | 12.5 | |
| qT1qF11qF17 | F081 | rep13 | 3 | |
| qT1qF11qF17 | F081 | rep14 | 6 | |
| qT1qF11qF17 | F259 | rep1 | 19 | |
| qT1qF11qF17 | F259 | rep2 | 3 | |
| qT1qF11qF17 | F259 | rep3 | 3 | |
| qT1qF11qF17 | F259 | rep4 | 0 | |
| qT1qF11qF17 | F259 | rep5 | 3 | |
| qT1qF11qF17 | F259 | rep6 | 0 | |
| qT1qF11qF17 | F073 | *NA* | *NA* | |
